# Short Talk on Protection

**Published:** 1881-06

**Authors:** 


### SHORT TALKS ON PROTECTION.

### PROTECTION, THE POOR MAN'S FRIEND.

[Issued by the Brooklyn Revenue Reform Club.]

The protective system could not stand a day if it were not believed by at least half the poor men of this country to be favorable to their interests. It would be amusing to observe how strongly this belief prevails among poor men, if it were not so melancholy to see them sacrificing their own comfort and that of their families to a gross delusion. What is protection? Nothing in the world except a heavy tax on the necessaries of life, imposed for the express purpose of making them dearer to ourselves. How is this tax paid? In proportion to men's lands, houses, property or incomes? No; but in proportion to what they spend upon themselves and their families. Mr. Vanderbilt is said to have an income of from $10,000,000 a year to any conceivable higher amount. But call it $10,000,000. He may possibly use imported goods to the extent of $50,000 a year; although we doubt it, except when he buys pictures, which do not pay much duty. But 1,000 of his workmen, earning $500 a year each, or $500,000 in all, will use a much larger amount of imported goods, paying an equal duty, although all of them together do not have an income one-twentieth part as large as his, and do not own one-hundredth part of his property. Now it is only fair to Mr. Vanderbilt to say that he does not especially support this system, much as he profits by it. His workmen, who generally pay nineteen-twentieths of his taxes, are much more bent upon keeping up this wonderful system than he is.

